# Buffalo Medical and Surgical Association

**Published:** 1882-05

**Authors:** 


### Society Reports.

### Buffalo Medical and Surgical Association.

*Annual Meeting, April 4, 1882.*

The Vice-President, Dr. A. R. Davidson, in the chair.

The subject for special consideration was presented in a paper by Dr. W. C. Barrett on "Protoplasm." He gave a review of the progress of biological research from the period of the introduction of the microscope down to the present day, and a resume of the more important truths which the labors of eminent histologists have given to the profession.

As to prevailing diseases Dr. Rochester said, that he thought there was an usual amount of sickness, but little mortality. Eruptive fevers, especially scarlet fever and measles were epidemic; German measles or roseola was also quite prevalent, much more so than ever before in his experience. The disease was found among persons of all ages and was generally light, but sometimes became complicated with diphtheria or other diseases and was more severe. Scarlet fever was very prevalent but not in a malignant form. Pleuro-pneumonia was met with very severe in character and often proving rapidly fatal.

Dr. Cronyn, Dr. Briggs and others also remarked upon the unusual prevalence of rötheln.

The association then proceeded to ballot for officers for the ensuing year, the result of which was the election of the following: President, A. R. Davidson, M. D.; Vice-President, John Cronyn, M. D.; Secretary, C. M. Daniels, M. D.; Treasurer, F. E. L. Brecht, M. D.; Librarian, J. B. Samo, M. D.

